# Modeling of an Inverted Drift Tube for Improved Mobility Analysis of Aerosol Particles

**DOI:** 10.1038/s41598-017-06448-w

**Published:** 2017-07-25

**Authors:** Minal Nahin, Derek Oberreit, Nobuhiko Fukushima, Carlos Larriba-Andaluz

**Affiliations:** 1 Integrated Nanosystems Development Institute(INDI), IUPUI, Department of Mechanical Engineering, Indianapolis, IN 46106 USA; 2 Kanomax FMT Inc., St. Paul, MN 55110 USA; 3 Kanomax Japan Inc., Shimizu, Suita-shi, Osaka 565-0805 Japan

## Abstract

A new mobility particle analyzer, which has been termed Inverted Drift Tube, has been modeled analytically as well as numerically and proven to be a very capable instrument. The basis for the new design have been the shortcomings of the previous ion mobility spectrometers, in particular (a) diffusional broadening which leads to degradation of instrument resolution and (b) inadequate low and fixed resolution (not mobility dependent) for large sizes. To overcome the diffusional broadening and have a mobility based resolution, the IDT uses two varying controllable opposite forces, a flow of gas with velocity *v*_*gas*_, and a linearly increasing electric field that opposes the movement. A new parameter, the separation ratio *Λ* = *v*_*drift*_/*v*_*gas*_, is employed to determine the best possible separation for a given set of nanoparticles. Due to the system’s need to operate at room pressure, two methods of capturing the ions at the end of the drift tube have been developed, Intermittent Push Flow for a large range of mobilities, and Nearly-Stopping Potential Separation, with very high separation but limited only to a narrow mobility range. A chromatography existing concept of resolving power is used to differentiate between peak resolution in the IDT and acceptable separation between similar mobility sizes.

## Introduction

Charged gas phase nanoparticles can be subject to drift and separation by means of electric fields. The charge divided by the friction coefficient of the nanoparticle under such fields is defined as electrical mobility and its accurate reckoning is key to the determination of particle size distribution functions. In Aerosol Science, and when dealing with globular particles, a particle’s electrical mobility is linked to its diameter, *d*_*p*_, through the well-known equation^1, 2^:

1 $$Z=\frac{qe{C}_{C}({K}_{n},\lambda ,{d}_{p})}{3\pi \mu {d}_{p}}$$

where *qe* is the net charge on the particle (the product of the integer charge state and the unit electron charge), *µ* is the dynamic viscosity and *λ* is the mean free path. *C*_*c*_ is the Cunningham’s correction factor and is a function of the Knudsen number. Most often, mobility based size distribution functions are measured with differential mobility analyzers (DMA)^3^ coupled to Condensation Nucleus Counters CNCs^4^, and operated in series as a scanning mobility particle sizer (SMPS)^5, 6^. While the SMPS combination has been incredibly successful, there are several shortcomings to its use which could be improved upon employing different techniques. Because the residence time -length divided by sheath velocity- of transmitted particles in a DMA is fixed and independent of particle size, diffusional broadening leads to degradation of instrument resolution for sub 20 nm particles^7, 8^. For particles larger than 20 nm, the resolution, defined as *Z*/Δ*Z*, is fixed and with values of approximately <10 for most operating commercial devices. This results in adequate resolution but sometimes insufficient -a 90 nm monodisperse distribution is barely distinguishable from a 100 nm monodisperse distribution assuming a resolution of 6-. Similarly, SMPSs typically require several minutes to complete voltage scans^5, 6^, and even in faster scanning instruments, particles of different sizes are sampled at different times. This limits information that can be obtained when aerosols are varying rapidly, such as can occur during sampling with an aircraft, near roadways, or from a combustion engine. Further, DMAs require the use of high sheath flow rates, and as such, require modest to high flowrate pumps which must remain stable during operation. This, along with the need of a scanning high voltage source, leads to increase costs in DMA operation. For mobility spectrometers at atmospheric pressure that deal with nanoparticles between 1–120 nm, there is therefore the need to 1) increase resolution by correcting diffusion broadening of nanoparticles in the drift cell, 2) increase the maximum fixed resolution or make the resolution directly proportional to particle size (or inverse mobility) and 3) obtain complete unsteady profiles of particles on rapidly varying aerosols.

Problems 2 and 3 have been resolved somewhat by the use of Drift Tube Ion Mobility Spectrometers (DT-IMS)^9^. In such systems, particles of all mobilities are sampled as a packet at a specific time and are guided by a constant electric field to the detector providing separation that depends on the length and electric field^10^. Particles are however still affected by diffusion broadening and, for a fixed electric field and length, yield fixed resolutions independent of size. To achieve high resolution (>100), the instrument length must be on the order of meters or make use of fairly high electric fields^11, 12^, precluding its use as a portable instrument to measure 20–100 nm particles. The need to improve the Ion Mobility systems is quite apparent as multiple improvement designs are becoming available in the last years such as the ROMIAC, the UMN DTIMS or the FIMS^13–17^ at room pressure or the SLIM or Trapped Ion Mobility (TIMS) instruments^18, 19^ at low pressures.

Here a new atmospheric pressure instrument is proposed that is based on the DT-IMS, but which uses two varying controllable opposite forces to correct for diffusion broadening while having its resolution be dependent on mobility, and which increases with the size of the particle. The instrument has been termed Inverse Drift Tube (IDT) due to the electric field being opposed to the migration of the nanoparticles. The concept of using opposing forces is not new as was used by John Zeleny^20^ in his experiments dating back as far as 1898. It is also the same principle that is used in the more recent TIMS spectrometer. However, the merit of the TIMS lies in the use of RF to confine the ions at low pressures^21^. This RF confinement is not possible at atmospheric pressures and thus TIMS is not usable at pressures where the IDT is intended. It also precludes its portability. Therefore, other means of collecting the nanoparticles must be employed in the IDT where the ions cannot be trapped.

The text is divided into the characterization of the IDT, followed by a theoretical explanation of the system and its resolution, and ending with 1D and 3D simulations of nanoparticles. In particular, 3D simulations are performed using SIMION, where flow and electric fields are fully determined, and where stochastic diffusion simulations are employed to calculate accurate distributions of nanoparticles.

## Methods

### Characteristics of the Inverted Drift Tube

#### Shortcomings of previous drift instruments. Resolution of the DMA and Drift Tube

The main concept of the proposed device is to be able to increase the resolution of the existing instruments for a broad range of sizes (<1 nm–120 nm in diameter) while maintaining sensitivity (comparable to 20 cm drift tubes). In order to improve resolution, one can try to overcome the shortcomings of the DMA and Drift Tube. The ideal non-diffusional transfer function of a DMA is a well-known function of the ratios of the flow rates. In terms of resolution, one can write^22^:

2a $${R}_{DMA}=\frac{{Q}_{sh}+{Q}_{e}}{{Q}_{a}+{Q}_{s}},$$

where *Q*_*sh*_ is the incoming sheath flow rate, *Q*_*e*_ is the excess output flow rate, *Q*_*a*_ is the aerosol inlet flow rate and *Q*_*s*_ is the aerosol outlet flow rate. The resolution is rarely higher than 10 -although theoretically it could reach values of up to 100 for large sheath to aerosol flow ratios- and remains fixed for all mobilities. Although not appearing in eq. (2a) the DMA resolution is strongly affected by the diffusion broadening effect. The diffusional variance is in inverse proportion to the voltage ${\sigma }_{diff}^{2}\propto 4kT/qV$ ^23^ which will greatly affect higher mobility particles (smaller diameter particles) since the voltage needed to filter them through the DMA is smaller yielding lower values of resolution for highly mobile particles.

Given that DT-IMS separation occurs in time -by allowing the ions to travel a given length-, its resolution, $\bar{x}/{\rm{\Delta }}x$, is given by^10^:

2b $${R}_{DT-IMS}=\frac{\bar{x}}{{\rm{\Delta }}x}=\frac{t{v}_{drift}}{{(16{D}_{L}tln2)}^{1/2}}={(\frac{{\boldsymbol{qEL}}}{16{\boldsymbol{kTln}}2})}^{\frac{{\bf{1}}}{{\bf{2}}}},$$

where *q* is the charge, *E* is the electric field, *L* is the tube length, *D*_*L*_ is the longitudinal diffusion coefficient and *T* is the temperature. As is evident, the resolution increases with the electric field and the length as the ½ power. It is also however important to note that the broadening of the peak *Δ* *x* is always affected by *D*_*L*_ *t* which increases with time. Many instances can be found in literature that attempt to increase resolution by increasing the length of the drift tube^24–26^. This however comes at the cost of sensitivity losses or appearance of artifacts. The DT-IMS has resolutions that are normally close to 100 for 2 meter instruments but they suffer from diffusion broadening and are not mobility dependent. Large electric fields could be used to obtain higher resolutions and smaller instruments. However, for portable instruments (~10 cm) and particles of fairly low mobilities (K > 1e-8 m2/Vs), the required electric fields (40 kV/m) to have resolutions >100 would yield acquisition times of 250 s that most likely would lead to the loss of the nanoparticles prior to reaching a detector.

Accordingly, there is a need to overcome, stop or delay the diffusion broadening effect that affects both DMAs and Drift Tubes while having a resolution that scales with inverse mobility. A plausible idea is to try and mimic the advancing of the ion through a drift tube without the need to cover any length by providing a second force that restricts the advancing of the ions while maintaining mobility separation -hence gaining the drift tube advantage of length dependent resolution without the need of long tubes-. In the IDT, one can make use of the electric field as an opposing mechanism to the advancement of the ions through the cell. This still allows for separation in time but where the mobility separation is no longer only subject to a direct square root dependence of the length and field.

#### A sketch of the Instrumental Inverted Drift Tube (IDT)

**Figure 1:**
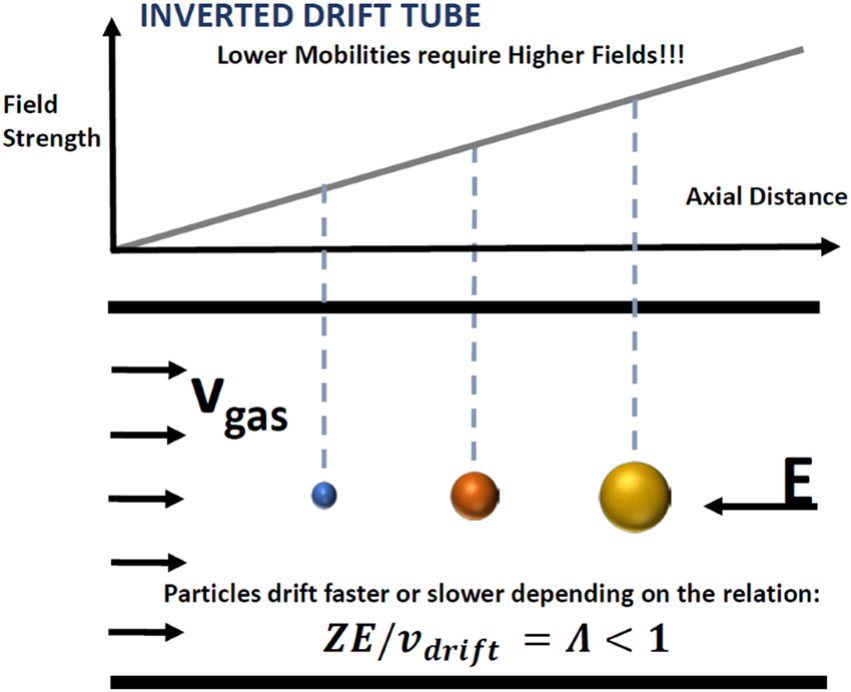
Sketch of the Inverted Drift Tube System. Note the gas flow in the direction of the moving ions and the linearly increasing electric field in the opposite direction.

Concept is shown in Fig. 1. The instrument consists of a 120 mm long tube and 48 mm diameter making the system a compact drift cell when compared to regular Drift Tubes. The separation mechanism is as follows. At room pressure, packages of ions of multiple mobilities, inserted at the entrance of the tube at time *t*_0_ using a pulsed Bradbury-Nielsen gate, are pushed by a gas flowing with a velocity *v*_*gas*_ downstream. A series of electrodes are equally spaced inside the drift tube and are connected through resistors into a power supply. The electrodes are used to create a linearly increasing electric field which opposes the gas flow of the ions slowing their movement relative to *v*_*gas*_(~0.04 m/s unless otherwise specified). This allows the ions to be separated depending on their mobility through the drift velocity *v*_*drift*_ = *KE*. Given that the field opposes the flow, the lowest mobility ions are the ones ahead in contrast to a regular DT-IMS hence the term “Inverted”. As long as the ratio $\frac{{v}_{drift}}{{v}_{gas}}$, termed from here on *separation ratio*, *Λ*, is smaller than unity, the ions of a given mobility will traverse through the drift cell without being completely stopped. Eventually, these ions can be collected downstream of the drift tube. The closer $\Lambda =\frac{{v}_{drift}}{{v}_{gas}}$ is to 1, the longer time the ion remains in the drift cell and the higher the separation will be. However, if at some point inside the drift cell, the ratio *Λ* = 1 is reached, particles of such mobility would be stopped and pushed towards the walls due to the residual radial electric field that arises from Laplace’s equation where ∇^2^ *V* = 0 (see section 3). Due to the existing room pressure, confining the stopped ions using an RF is quite difficult and will not be pursued.

With a large range of ion sizes, the main idea is therefore to allow all mobilities of interest to traverse the drift cell while trying to keep the ratio *Λ* as close to 1 as possible without losing the ions. As *Λ* is mobility dependent, not all mobility ranges being analyzed simultaneously will have high separation ratios. With that in mind, two different mechanisms have been explored to separate ions using the IDT:

1. *Intermittent Push Flow(IPF)*: When trying to separate a wide range of mobilities, a need to vary the opposing electrical field is necessary to acquire all mobilities at high resolutions. The method is depicted in Fig. 2a where the highest possible field slope (*dE*/*dx* = *A*) for a given electrical supply is initially selected. Before the ion with the highest mobility hits a separation ratio of *Λ* = 1, the slope of the electric field is lowered and thus ensuring that *Λ* = 1 will never be reached. This drop in the slope can happen as many times as needed until all necessary ions are collected at the end of the drift tube. The resolution and separation of the peaks will depend on the range of mobilities.

**Figure 2:**
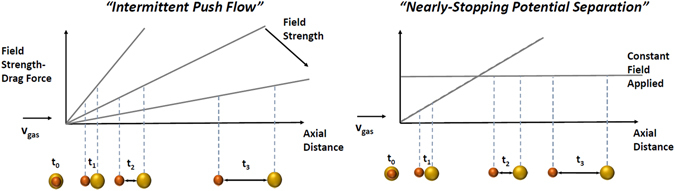
(**A,Left**) Intermittent Push Flow. Ions or charged particles are separated by subsequently lowering a linearly increasing field opposite to the flow. (**B,Right**) Nearly-Stopping Potential Separation. An opposite constant field with separation ratio below 1 is used to maximally separate two relatively close mobilities.
2. *Nearly*-*Stopping Potential Separation (NSP):* When trying to separate ions of very close mobilities, an alternative possibility is to use an opposing “constant” electric field which is slightly below the necessary field to maintain the separation ratio slightly below 1 for all ions of interest hence maximizing the separation potential. This method is depicted in Fig. 2b.

Whether one mechanism or the other is used will depend on the range of mobilities and the resolution required. The advantage of these type of opposing-field instruments is their autocorrecting properties when used as *Intermittent Push Flow* separators. In the next section, a derivation of the transport distributions for ions in the IDT with linearly increasing fields is analytically established focusing on its autocorrecting properties and instrument resolution.

While different versions of instruments (such as Trapped Ion Mobility Spectrometers(TIMS),^27^) use electric fields opposite to the flow and have been applied in recent years, the implementation has always been at low pressure and for very high mobilities, which allows the particles to be captured using an RF field when $\frac{{v}_{drift}}{{v}_{gas}}=1$, something that cannot be pursued at room pressure. After *Λ* = 1 has been reached in TIMS, the ions are then subsequently pushed by lowering the electric field to the critical value that pushes the particles through. Due to the difficulties of operating RF at room pressure, its implementation and the main principle of trapping the ions used by TIMS will not be pursued in this work.

## Results

### Theory

#### One Dimensional Transport of Species in the gas phase migrating through a linearly increasing opposed electric field. Resolution of an Inverted Drift Tube

To simplify the picture, we will assume that the IDT has a fixed increasingly linear electric field to study particles of only one mobility. Let a concentration of *n*(*r*, *z*, *t*) ions of charge *q* drift through a tube where a gas flows at a velocity *v*_*gas*_ and where a linearly increasing electric field, $\mathop{E}\limits^{\longrightarrow}\,=Az\vec{k}$, is applied opposite to the flow (*A* is the slope of the field). In a case where the concentration of ions *n*(*r*, *z*, *t*) is low enough that space charge can be neglected, the balance equation for the species can be given by:

3 $$\frac{\partial n}{\partial t}-\nabla \cdot (\bar{\bar{D}}\cdot \nabla n-({\vec{v}}_{gas}-K\vec{E})n)=0,$$

with $\bar{\bar{D}}$ being the diffusion tensor and *K* the electrical mobility. If one considers the one-dimensional problem neglecting radial electric field and diffusion, eq. (3) can be written in Cartesian coordinates as:

4a $$\frac{\partial n}{\partial t}={D}_{L}\frac{{\partial }^{2}n}{\partial {z}^{2}}-({v}_{gas}-KAz)\frac{\partial n}{\partial z}+KAn,$$

being the initial concentration at time *t*_0_ at the beginning of the tube the surface density:

4b $$n(0,0)={n}_{s}$$

The Sturm-Liouville solution to the equation for the aforementioned initial concentration can be written as:

5-1 $$n(z,t)=\frac{{n}_{s}}{\sqrt{2\pi {\sigma }^{2}}}{e}^{-\frac{{(z-\bar{x})}^{2}}{2{\sigma }^{2}}};with$$

5-2 $${\sigma }^{2}=\frac{2{D}_{L}}{{v}_{gas}}(\bar{x}-\frac{KA}{2{v}_{gas}}{\bar{x}}^{2})=\frac{{D}_{L}}{KA}(1-{e}^{-2KAt})=\frac{kT}{qA}(1-{e}^{-2KAt});and$$

5-3 $$\bar{x}=\frac{{v}_{gas}}{KA}(1-{e}^{-KAt})$$

There are several features that differentiate this equation from that of the regular drift tube. Most importantly the standard deviation *σ* as shown in equation (5-2) has a correction term $\frac{KA}{2{v}_{gas}}{\bar{x}}^{2}$. This “auto-correction” term is quadratic with the mean position $\bar{x}$ of the distribution so that it increases with the traversing distance through the drift cell. While the conventional Drift Tube distribution broadens as a function of time, the Inverted Drift Tube distribution broadening is damped and eventually stopped. The contribution of $\frac{KA}{2{v}_{gas}}{\bar{x}}^{2}$ increases with time (or distance) and, as the ion advances through the drift cell, invariably leads to an asymptotic value of the standard deviation given by *kT*/*qA* and independent of t (see eq. 5-2). This asymptotic value will be reached when the separation ratio *Λ* becomes one. In such instance, the mean position of the distribution will be given by $\frac{{v}_{gas}}{KA}$ and the ion will no longer advance in the axial direction.

The resolution for the IDT can be calculated from eq. (5) as:

6 $$R=\frac{\bar{x}}{{\rm{\Delta }}x}=\frac{\bar{x}}{2\sqrt{2\,\mathrm{ln}(2)\frac{kT}{qA}(1-{e}^{-2KAt})}}$$

If the drift cell has length L, the resolution at distance L can also be calculated as:

7 $${R|}_{L}=\frac{L}{\sqrt{16\,\mathrm{ln}(2)\frac{KkT}{q{v}_{gas}}(L-\frac{KA}{2{v}_{gas}}{L}^{2})}}=\frac{\sqrt{qL}}{\sqrt{16\,\mathrm{ln}(2)\frac{KkT}{{v}_{gas}}(1-\frac{KA}{2{v}_{gas}}L\,)}}=\frac{{R}_{DT-IMS}}{\sqrt{{\rm{\Lambda }}(1-\frac{{\rm{\Lambda }}}{2}\frac{L}{z}\,)}}$$

**Figure 3:**
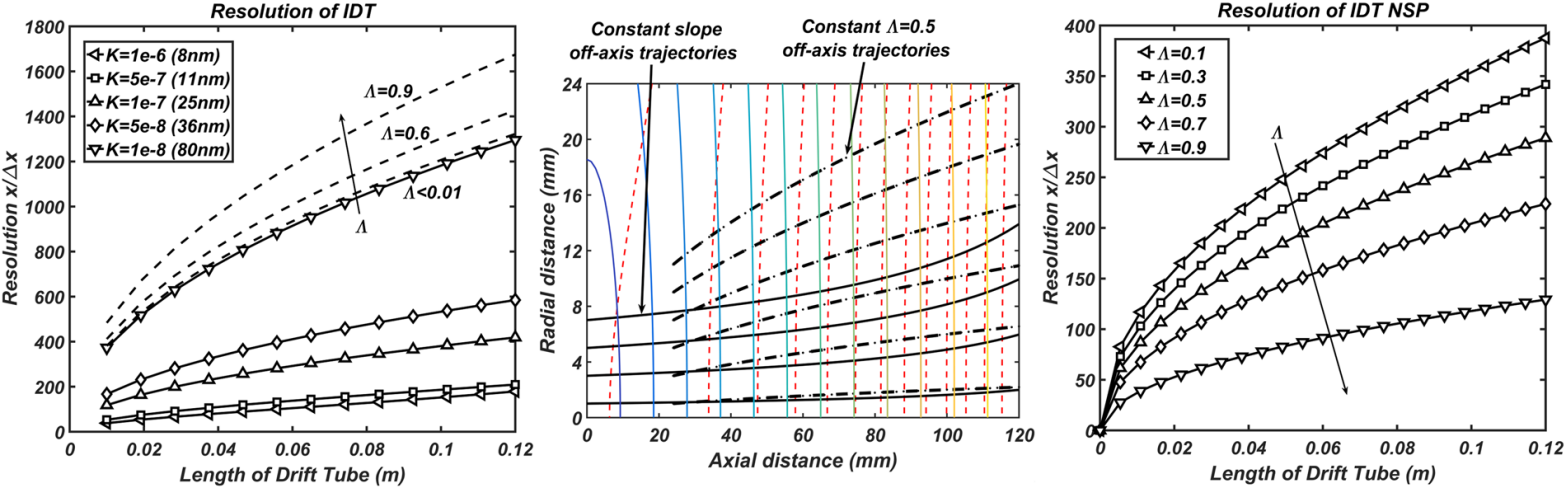
(**A,Left**) Mobility dependent IDT Resolution for *v*_*gas*_ = 0.04 *m*/*s* and *A* = 3.2*e*5 *V*/*m* ^2^ for Intermittent Push Flow(IPF). Dashed lines show theoretical maximum when keeping the separation ratio constant. (**B,Center**) Off axis trajectories due to the existing radial field in Intermittent Push Flow. Perpendicular lines to the axial direction are isofield lines (colormap) and isopotential lines (dashed red) for a constant A. Solid black lines correspond to off axis trajectories at different initial radial conditions using Intermittent Push flow for constant slope A. Dotted black lines correspond to trajectories at a constant separation ratio *Λ* (**C,Right**) IDT resolution using the Nearly-Stopping Potential (NSP) method (eq. 8).

Figure 3A shows the resolution as a function of length and different mobilities for a fixed slope *A* and *v*_*gas*_ (solid lines). Note that unlike the resolution of the Drift Tube depicted in eq. (2b), the resolution of the IDT has a positive dependence on the mobility. The resolution of the instrument is very high in terms of eq. (7) for very modest lengths, but, given the large difference in mobilities appearing in Fig. 3A (8–80 nm), the separation ratio is very small for the smallest mobilities. In fact, to avoid losing ions of any type, the separation ratio *Λ* is well below 1 for most of the length of the drift tube (it is only close to one for the 8 nm particles when it reaches 12 cm and it is 0.01 for the 80 nm particle at 12 cm). To improve the resolution and separation ratio, one must resort to the tactics used in the previous section, i.e. use of IPF and/or NSP separation. For the intermittent push flow, there is a theoretical optimal resolution for which the separation ratio *Λ* is kept constant for a particular mobility. This requires continuous change of the field slope A to guarantee that at any given position of the ion *Λ*~*constant*. Figure 3A shows the theoretical resolution maximum (dashed lines) for 80 nm particles as a function of the different constant values of the separation ratio. The third expression in eq. (7) is obtained by using eq. (2b) and the definition of the separation ratio. Resolution as given in eq. (7) is not particularly useful for this type of instrument as opposed to the DMA or DT-IMS. The reason is that if the field slope was A = 0, i.e. no electric field, the residence time in the drift tube will be minimal and there will be no separation between any mobilities. However, the resolution would depend on the competition between diffusion and *v*_*gas*_ which could still be very high. The importance of eq. (7) relies on the fact that when the separation ratio *Λ* increases, the residence time inside the system increases, but the resolution also increases in contrast to what is expected with just diffusion.

Since the electric field is solenoidal when space charge is neglected, the radial electric field might be non-negligible off axis and one must wonder about its effect in the trajectories of the ions as the separation ratio is increased. Given $\nabla \cdot \vec{E}=0$, one can calculate the radial field for constant axial field cross sections $(\frac{\partial {E}_{z}}{\partial z}=-A)$ and which is given by *E*_*r*_ = *Ar*/2. Figure 3B shows the trajectories of off-axis particles due to the effects of radial electric fields (no diffusion considered) for two particular cases: a) constant separation ratio *Λ* = 0.5 (dashed lines) and b) constant slope *A* = 2.5*e*5 and ion mobility $K=1\cdot {10}^{-6}{m}^{2}/Vs$ (8 nm) (solid lines). For case a) the initial condition is set at z = 2.4 cm as the slope A would be too high to maintain a constant separation ratio *Λ* for smaller values of z. Values that are off axis up to 1.1 cm still reach the end of the tube. In case b), the separation ratio *Λ* increases with the distance z progressively reducing the velocity of the moving ions (*v*_*m*_ = *v*_*gas*_ − *v*_*drift*_) until the drift from the radial field becomes of the order of *v*_*m*_ at a given radial position. If this occurs, the ion will inevitably be lost. One must make note that the resolution might be space charge dependent when a high charge concentration is present. However, for small concentrations of large singly charged nanoparticles, this effect is expected to be negligible.

#### Resolution using constant opposed field (Nearly-Stopping Potential Separation)

To avoid radial field effects, one can resort to NSP separation mode where the electric field is constant. For such a case, the resolution of a distribution of ions, *R*_*NSP*_, of mobility *K* after passing through the cell is equivalent to that of a DT-IMS (eq. (2b)) but where the field opposes movement:

8 $${R}_{NSP}=\frac{\bar{x}}{{\rm{\Delta }}x}=\sqrt{\frac{qL{v}_{m}}{16kTln(2)K}}=\sqrt{\frac{qL{v}_{gas}(1-{\rm{\Lambda }})}{16kTln(2)K}}$$

Note how in this case, the separation ratio opposes resolution. The reasoning is quite clear, the larger the separation ratio, the longer the total residence time in the drift tube and the higher the chance ions have to diffuse before covering a distance L. Figure 3C shows the resolution for nearly stopping potentials for a given mobility $K=1\cdot {10}^{-7}{m}^{2}/Vs$ (25 nm) as a function of the separation ratio *Λ*. It is clear form eqs (7, 8) that $R=\bar{x}/{\rm{\Delta }}x$ is an ill-conditioned term to define ion separation for the IDT instrument since, as the separation ratio increases, mobility separation in time should be more likely opposite to what the resolution predicts. It is necessary to resort to a different criterion to establish whether ions of different mobilities can be separated and how well. In section 3.2.3, resolving power (*R*_*p*_) will be introduced to account for mobility separation. Section 3.2 will develop numerical simulations that (a) show the effect of autocorrection for a 1D simulation and (b) show separation capabilities in a 3D simulation with stochastic diffusion simulation using SIMION.

### Numerical Simulations

#### 1-D Numerical simulation with diffusion autocorrection

**Figure 4:**
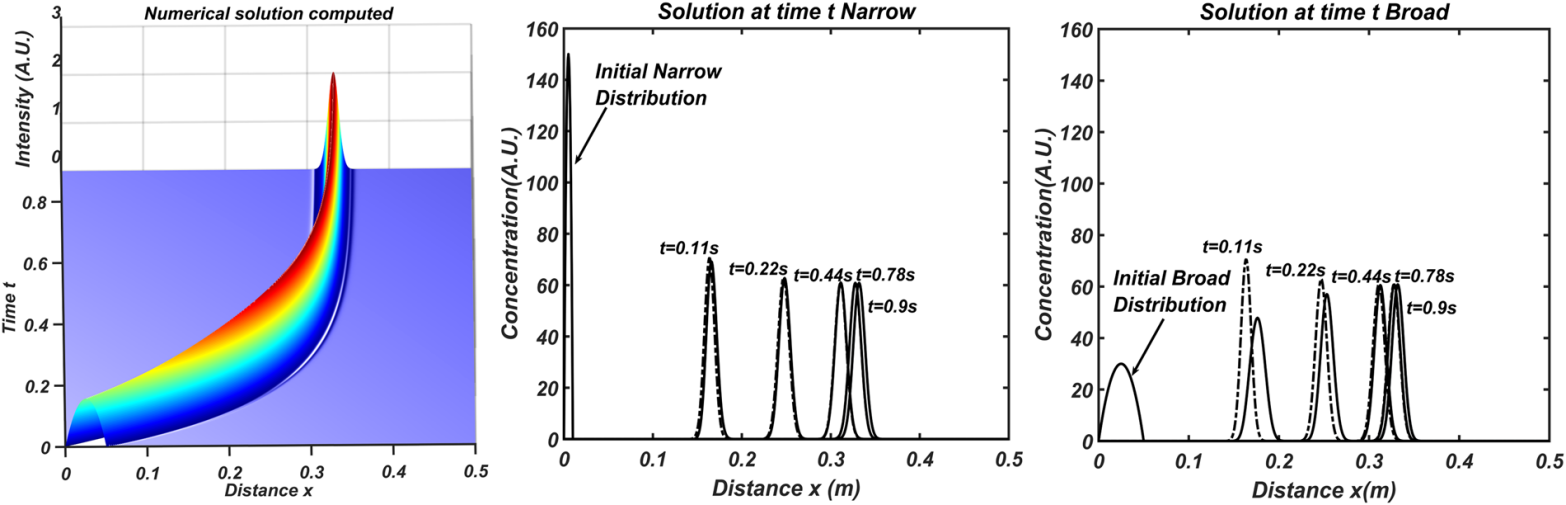
(**A,Left**) 1D numerical solution for eq. (2) showing the position of the distribution as a function of time for an initial broad parabolic distribution *n*_*s*_(*χ*). (**B**,**C**) Simulation of the 1D IDT eq. (2) at different times. Dashed lines correspond to the analytical solution eq (5-1) while solid lines correspond to the numerical solution at times t = 0s, 0.11s, 0.44s, 0.78s and 0.9s for two cases. (**B,Center**) Initial narrow distribution. (**C,Right**) Initial broad distribution. Diffusion autocorrection is able to narrow down the initial distribution as it travels through the drift cell.

1D numerical solution of equation (2) is shown in Fig. 4A where the position of the distribution of singly charged ions of a single mobility is given as function of time for a specific set of parameters, namely *A*,*K*,*v*_*gas*_,*D*_*L*_ and initial condition *n*_*s*_(*x*). The advantage of using numerical methods is that one can easily use the initial condition to be a distribution *n*_*s*_ of any kind at time *t* = 0 and study its evolution. On Fig. 4A there are a couple of notable features. The first of them is that there exists a maximum value of x at which the distribution of ions reach an asymptotic behavior. This asymptotic behavior occurs, as stated previously, at ${\bar{x}}_{asympt}=\frac{{v}_{gas}}{KA}$ given by the separation ratio reaching a value of 1. The second feature is that the standard deviation of the ions will also asymptotically tend to a value *σ*_*asympt*_ = *kT*/*qA* regardless of the initial distribution. To test whether the asymptotic standard deviation was reached from different initial distributions, two initial distributions were modeled; one with smaller standard deviation than the asymptotic value (*σ*_*initial*_ < *σ*_*asympt*_) and one with a larger one (*σ*_*initial*_ > *σ*_*asympt*_). The distributions as a function of time are shown in Fig. 4B,C and both are compared to the analytical solution -obtained using eq. (5-1)- giving excellent agreement between numerical (solid) and analytical (dashed). Figure 4B clearly shows that when the initial distribution is very narrow, the distribution broadens and reaches the expected asymptotic behavior. The behavior follows the analytical solution quite accurately. When the initial distribution is broader than the expected asymptotic solution as in Fig. 4C, the distribution narrows in order to reach the expected asymptotic solution. This behavior is unique to the IDT instrument and provides the possibility of ultra-high resolution if we allow the ions to stay in the cell for a sufficient length/time. The reasoning behind the autocorrection of the diffusion broadening is quite apparent. Ions diffusing to the left of the equilibrium point have values of *Λ* < *Λ*_*eq*_, and are pushed forward faster than those at *Λ*_*eq*_. Similarly, for those ions diffusing to the right, *Λ* > *Λ*_*eq*_, and they suffer a stronger electrical field which subsequently pushes them back to equilibrium.

#### 3D Statistical Diffusion Simulation IDT using SIMION for ion trajectories

**Figure 5:**
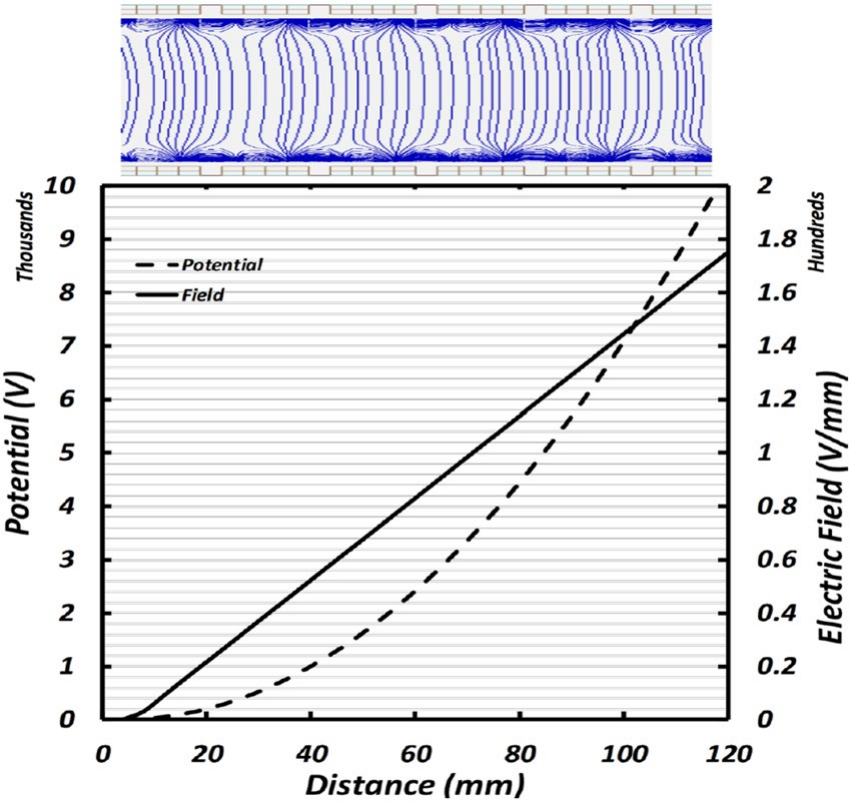
Top- Schematic of the IDT. Constant electric field lines are shown for convenience (blue). Bottom.- Expected axial voltage and field due to the electrodes.

To fully test the validity of our IDT system for single particle trajectories in a full 3D environment, we opted to use the commercial software SIMION. SIMION is an ion optics simulation software package that can model ion trajectories using three-dimensional electrostatic potential arrays. SDS (Statistical Diffusion Simulation) is SIMION’s internal subroutine that simulates the motion of charged particles in electrostatic fields under atmospheric pressure. In SDS, ion motions are simulated using a combination of viscous drag forces and random ion jumping that depends on the number of collisions of ions with gas molecules simulating Brownian diffusion. In order to completely simulate gas flow field lines, a CFD simulation of the drift tube using Fluent was undertaken and the velocity profile tabulated and used in SIMION. Figure 5 shows a section of the IDT with simulated electric field lines (blue) for the axial potential and field lines showed to the right of Fig. 5. This field lines agree with those presented in Fig. 3B for the analytical solution. The existence of this radial field causes ions with separation ratios *Λ* close to 1 to be pulled towards the electrodes and precluding the possibility of capturing the particles inside the drift tube. To avoid reaching separation ratios very close to 1 the two techniques mentioned in section 2 were developed and run in SIMION: Intermittent Push Flow (IPF) and Nearly-Stopping potential (NSP) separation.

**Figure 6:**
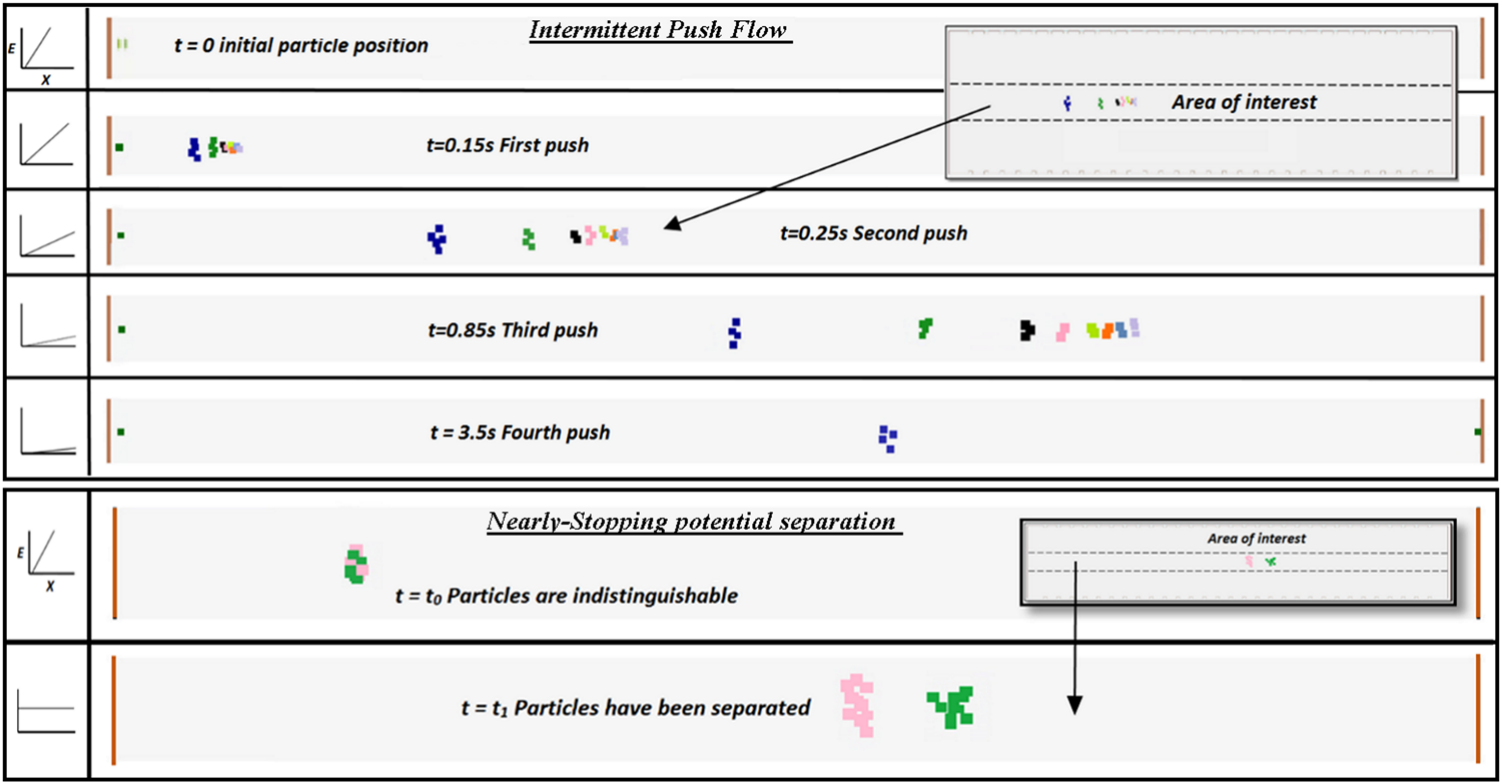
(**A,Top**) SIMION simulations of particles ranging from 20 to 120 nm in diameters using intermittent push flow. Simulations are shown at the time when each of the subsequent pushes is taken by lowering the slope of the field. All particles are easily separated in time and space and collected at the end of the drift tube. (**B,Bottom**) Simulation of Nearly stopping potential separation method depicted in Fig. 2b for two particle sizes of 55.89 and 55.93 nm where both particles have been separated.

**Figure 7:**
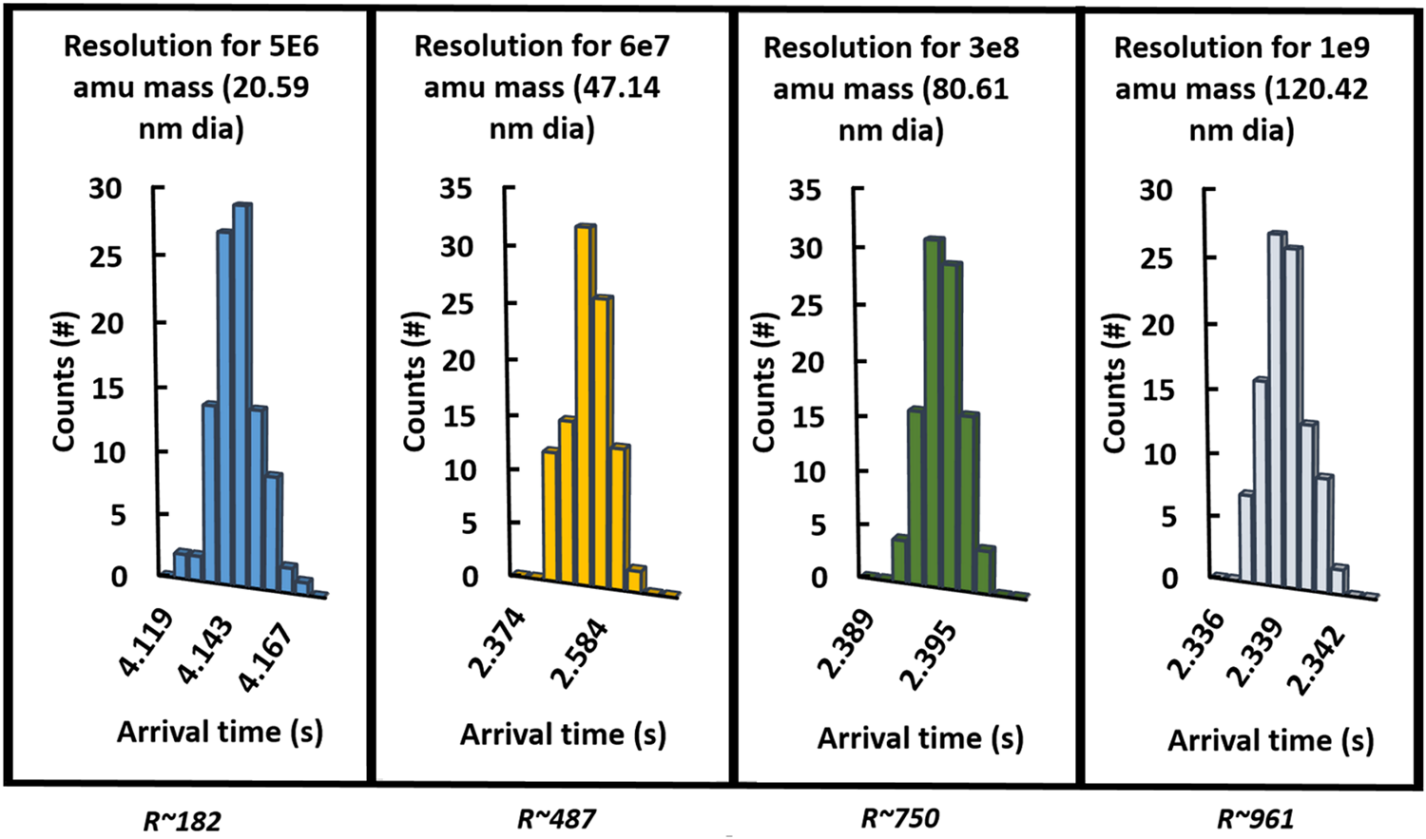
Distributions and Resolution *R* ~ *t*/Δ*t* of simulations using the set of intermittent pushes shown in Fig. 6 for different particles sizes. 100 nanoparticles were used to create the distributions.

Intermittent Push Flow Simulation: The first technique carried out is the IPF approach where the field slope is lowered at known fixed times so as to never reach a separation ratio of *Λ* = 1. Figure 6A shows a SIMION simulation at 5 particular times using model spherical nanoparticles of 8 different sizes ranging from 20 to 120 nm as they travel through the inverted drift tube. The times shown coincide with those used to lower the values of the electric field slope. To optimize the pushes, the pushes are made right before the separation ratio *Λ* becomes 1 for the highest mobility particle (blue in the figure). Using this method, all particles are collected and easily separated in the span of 4.2 seconds. However, the largest particles -those with separation ratio much smaller than unity- will not have achieved its maximum possible separation. It is conceivable however that if one was interested in resolving the largest particles, a higher separation ratio for such particles would be used, at the cost of losing higher mobility particles. For the pushes used in Fig. 6, 100 particles of each of the sizes studied were sampled and their time distributions collected in Fig. 7. The resolution was subsequently calculated as $\frac{t}{{\rm{\Delta }}t}$ and shown in the figure. The resolutions obtained are in agreement with those obtained in Fig. 3 and are extremely high compared to other instruments. Not a single particle was lost in the calculation process so the transmission was 100%. However, the simulation had the starting particles initially centered in the drift tube (deviation was always less than 0.5 cm) and space charge was neglected for the calculations. In any case, the loss of nanoparticles to the walls, even when space charge is considered, is not expected to be much higher than that of existing commercial instruments when the initial distribution is centered (see Fig. 3B). Table 1 shows the mobilities and diameters of the particles used in the simulation as well as the electric field slope and the average positions of the ions when the pushes were made.

**Table 1:** Parameters used for the SIMION simulation for IPF.

Mass (amu)	Diameter (nm)	Mobility (m2/Vs)	Position of ions at time t (mm)			
			Push 1 at 0.15 s	Push 2 at 0.25 s	Push 3 at 0.85 s	Push 4 at 3.5 s
5.00E + 06	20.59	5.40E − 07	5.24	7.92	25.43	98.14
2.00E + 07	32.69	2.35E − 07	6.65	9.97	32.38	
6.00E + 07	47.14	1.22E − 07	7.17	10.85	36.04	
1.00E + 08	55.93	9.03E − 08	7.62	11.31	37.38	
2.00E + 08	70.42	6.03E − 08	7.51	11.61	38.45	
3.00E + 08	80.61	4.77E − 08	7.69	11.86	39.04	
5.00E + 08	95.57	3.56E − 08	7.84	12.59	39.39	
1.00E + 09	120.42	2.41E − 08	7.861	12.66	40.15	
**Electric Field Slope A (V/mm** ^2^ **)**			6.17	2.49	1.00	0.0.50

The average position of every type of ion is shown at the beginning of every push in mm.

Nearly-Stopping potential separation Simulation: For most of the sizes studied in section 3.2.2.1, the separation ratio was not near 1 and therefore the maximum expected resolution of the instrument was not achieved. To conceivably achieve the maximum possible resolution of the instrument for a given *v*_*gas*_ − *E* pair, the NSP method is used where the field applied is constant throughout the drift tube with a value that makes the separation ratio *Λ* close to 1 but slightly below for a nanoparticle of choice. Using a constant field eliminates the radial field component almost entirely and therefore separation ratios closer to 1 can be used without the risk of losing ions. Figure 6B shows a particular test where singly charged nanoparticles of 55.89 and 55.93 nm in diameter are trying to be separated (a 0.07% difference in diameter!) using this method. All nanoparticles with smaller diameters than 55.89 nm will have separation ratios larger than 1 and will be stopped at the entrance of the tube. All nanoparticles that are much larger than 55.89 will be promptly collected on the detector. As shown in the figure, initially both particles are indistinguishable. However, as they travel through the drift tube, eventually the 55.89 nm particle is contained inside the drift tube for a longer period of time and effectively separated from that of 55.93 nm. Even though the resolution given by eq. (8) decreases with *Λ*, the ability to separate ions in time increases with residence time inside the cell. Indeed, the effective comparable resolution for a 0.07% difference to occur in a regular DTIMS is in the thousands, something unimaginable with other systems. Such separation potential will have many applications in the aerosol and biochemistry fields. As an example, it could separate virus capsids of very similar sizes atomized in the air. To adequately describe the separation in terms of mobility and residence time, a new parameter, the resolving power, is defined in the following section.

#### Resolving Power vs. Resolution

**Figure 8:**
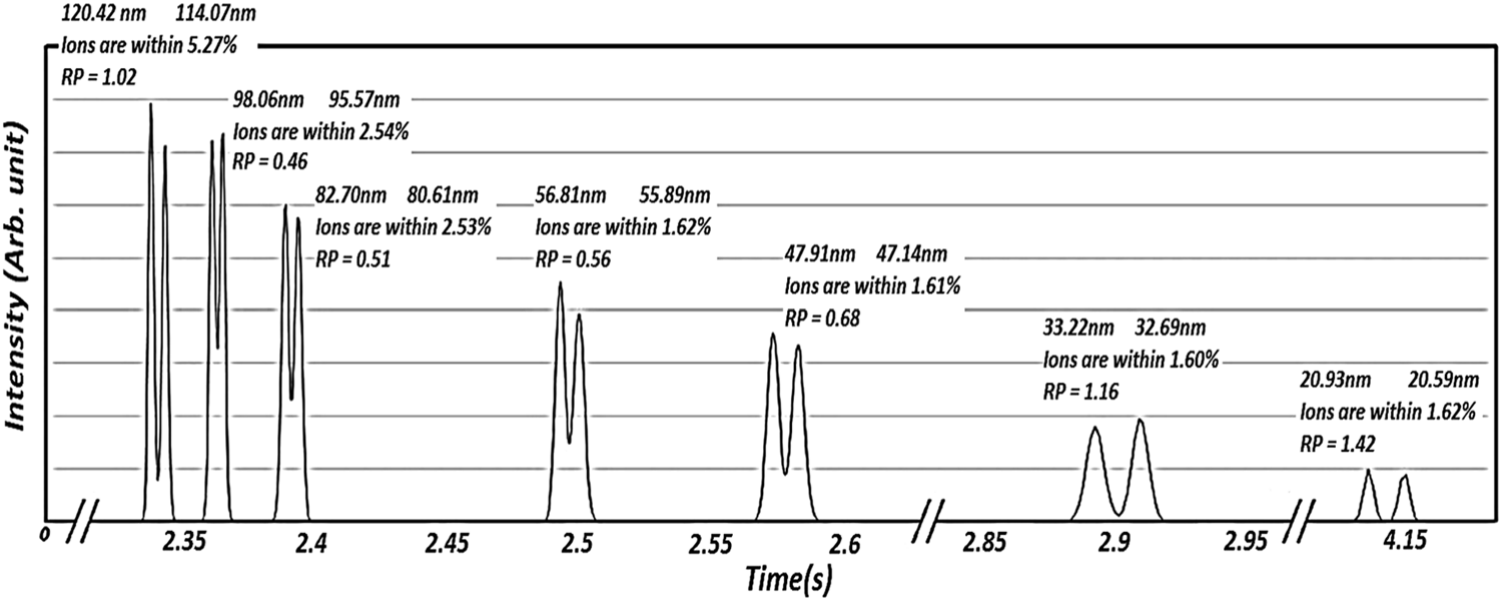
Separation of pairs of peaks using SIMION for two similar sizes. No optimization for resolution was attempted. Resolving power is used instead of resolution to specify whether or not two peaks are separable.

One would like to test whether or not the resolution provided by the IDT is sufficient to separate similar mobility particles using the intermittent push flow for a large range of sizes, i.e. 20 nm to 120 nm. Figure 8 shows simulations using SIMION of the distributions acquired when pairs of particles of very similar mobilities are separated inside the IDT for the same particular pushes given in Table 1. As can be observed, mobility separation is not directly related to resolution since ions of 20.59 nm and 20.93 and low resolution are at least as well separated, if not more, than the high resolution 114.07 nm and 120.42 nm ions. In order to describe if separation is possible as a function of residence time inside the drift cell, a better option to use instead is the resolving power employed in chromatography^28^, ${R}_{p}=\frac{{g}_{HW}}{W}$, given by the ratio of the gap between two peaks at the average half maximum, *g*_*HW*_, divided by the average width, *W*, of the peaks. From its definition, one should expect two peaks to be resolved if *R*_*p*_ > 0.1. The *R*_*p*_ values for the simulation are also shown in Fig. 8. Directly from the figure, one can tell that although the resolution for the initial peaks (particle diameter of 120.42 nm and 114.47 nm) is in the thousands, the fact that the separation ratio *Λ* for such ions is far from unity, provides only a resolving power *R*_*p*_ = 1.02 (enough to differentiate the peaks) even though the particle diameters differ by 6 nm. On the other hand, the last pair of peaks (20.59 nm and 20.93 nm), which have *Λ* closest to unity, have a *R*_*p*_ = 1.42 despite them being only 0.34 nm apart and while having resolutions of less than two hundred. It is clear that the time of residence inside the drift cell is key to separation. One can use the analytical resolution provided in equations 7 and 8 as a means to provide an analytical value of the resolving power. In such sense, *R*_*p*_ can be also defined the time difference between the arrival of the center of the distribution of two similar ions divided by the average FWHM of the two peaks minus one $({R}_{p}=\frac{{t}_{diff}}{\overline{FWHM}}-1)$. In particular, for IPF flow, and assuming a constant separation ratio through the drift cell, the resolving power is given by:

9 $$\begin{array}{rcl}{R}_{p(IPF)} & = & \frac{{t}_{1}-{t}_{2}}{1/2(FWH{M}_{1}+FWH{M}_{2})}-1\\  & = & \frac{\sqrt{qL{v}_{gas}}(\frac{1}{{v}_{m1}}-\frac{1}{{v}_{m2}})}{\frac{1}{2}\sqrt{16kTln(2)}(\frac{1}{{v}_{m1}}\sqrt{{K}_{1}(1-\frac{{{\rm{\Lambda }}}_{1}}{2})}+\frac{1}{{v}_{m2}}\sqrt{{K}_{2}(1-\frac{{{\rm{\Lambda }}}_{2}}{2})})}-1\end{array}$$

Similarly, for the Nearly-Stopping Potential separation:

10 $${R}_{p(NSP)}=\frac{\sqrt{qL}(\frac{1}{{v}_{m1}}-\frac{1}{{v}_{m2}})}{\frac{1}{2}\sqrt{16kTln(2)}(\frac{\sqrt{{K}_{1}}}{{v}_{m1}^{3/2}\,}+\frac{\sqrt{{K}_{2}}}{{v}_{m2}^{3/2}\,})}-1$$

**Figure 9:**
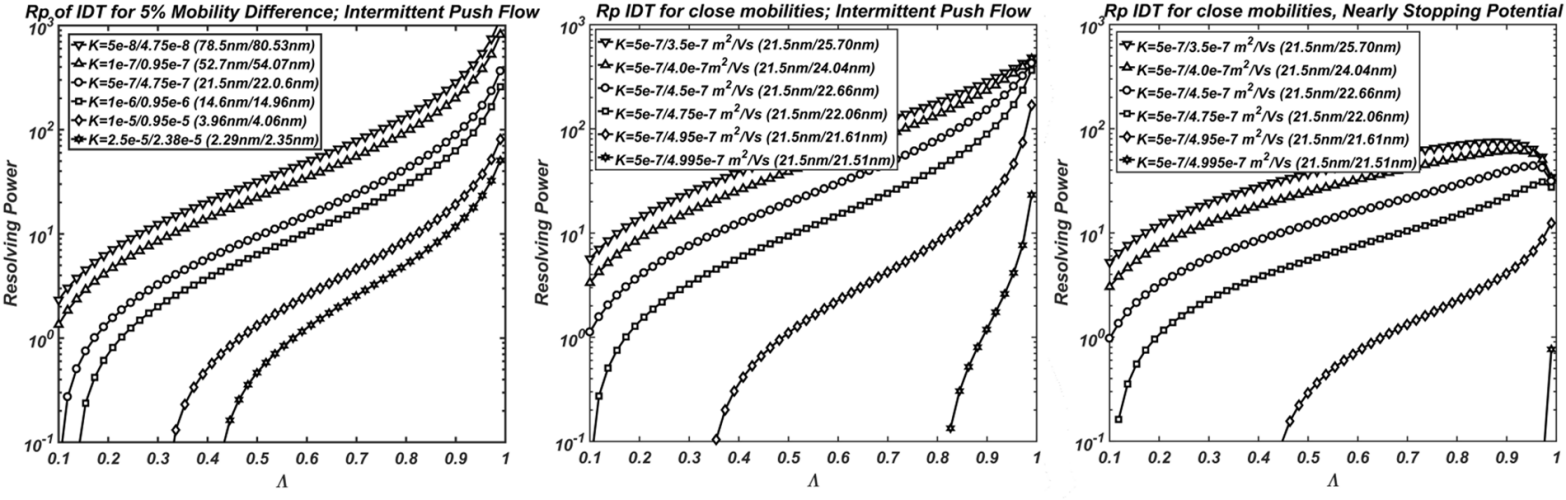
Resolving Power *R*_*p*_ as a function of separation ratio *Λ* for: (**A**) Ranges of ions with mobilities that differ 5% (2.23% in diameter) using Intermittent Push Flow. (**B**) An Ion with Mobility of 5e-7 m^2^/Vs (21.5 nm) with respect to ions that differ between 30% and 0.1% in Mobility for Intermittent Push Flow. (**C**) Same as (**B**) but for Nearly-Stopping Potential.

Figure 9 shows the resolving power as a function of the separation ratio *Λ* for different scenarios. Figure 9A shows the resolving power when trying to separate mobilities that differ 5% (2.23% in diameter) using the Intermittent Push Flow. The graphs are cut purposefully at *R*_*p*_ = 0.1 so that anything visible in the figure can be resolved at the appropriate separation ratio. In particular, lower mobilities are readily separable for any separation ratio, while higher mobilities require higher separation ratios. For example, to separate two ions that differ in diameter 0.06 nm at 2.29 nm requires at least a constant separation ratio of 0.43 or higher. To understand the capabilities of the IDT, Fig. 9B shows *R*_*p*(*IPF*)_ for nanoparticles that vary between 30% and 0.1% in mobility with respect to a value of 5e-7 m^2^/Vs (21.5 nm). Ions are easily separable up to 1% difference (21.5 nm to 21.61 nm) or less. However, to be able to separate a 0.01% one would require constant separation ratios of over 0.8. Due to the existing radial field, a separation ratio this high would most likely lead to the loss of the ions before reaching the end of the drift cell. In order to be able to use higher separation ratios, the nearly-stopping potential is used in Fig. 9C. The first thing to notice between Fig. 9B,C is that NSP requires higher separation ratios for the same mobility when compared to the IPF. This is due to the autocorrecting feature of the IPF disappearing when a constant electric field is used. This point can be made quite clear for low mobilities in Fig. 9C. While initially increasing the separation ratio, *Λ*, increases the resolving power, *R*_*p*_, this increase reaches a maximum at around *Λ* = 0.9 and then drops for larger values. At these very large residence times (large separation ratios), the diffusion velocity becomes of the same order or higher than the movement velocity *v*_*m*_ leading to a drop in *R*_*p*_. However, the fact that the NSP can be used at higher separation ratios makes it a strong candidate for separation when compared to the IPF.

## Discussion & Conclusions

A new mobility particle analyzer, which has been termed Inverted Drift Tube, has been modeled analytically as well as numerically and proven to be a very capable instrument. The basis for the new design have been the shortcomings of the previous ion mobility spectrometers, in particular (a) diffusional broadening which leads to degradation of instrument resolution and (b) inadequate low and fixed resolution (not mobility dependent) for large sizes. To overcome the diffusional broadening and have a mobility based resolution, the IDT uses two varying controllable opposite forces, a flow of gas with velocity *v*_*gas*_, and a linearly increasing electric field that opposes the movement. A new parameter, the separation ratio *Λ* = *v*_*drift*_/*v*_*gas*_, is employed to determine the best possible separation for a given set of nanoparticles. Due to the system’s need to operate at room pressure, two methods of capturing the ions at the end of the drift tube have been explored, Intermittent Push Flow for a large range of mobilities, and Nearly-Stopping Potential Separation, with very high separation but limited only to a very narrow mobility range. The following conclusions have been obtained:

1. Analytical description of the 1D IDT problem for an initial distribution of nanoparticles has been shown to yield very high resolutions without any optimization. Resolution is close to being proportional to the square root of the length, but has a dampening effect on the standard deviation that increases the resolution several folds when compared to a drift tube. The resolution is also proportional to the mobility. The lower the mobility, the higher the resolution. However, it is shown that the resolution is an ill-conditioned parameter to express whether or not separation occurs inside the drift cell.
2. A 1D numerical simulation of the IDT shows that there is an asymptotic value to the standard deviation for the intermittent push flow method. Regardless of the starting distribution, whether broad or narrow, the asymptotic behavior is achieved. The IDT has autocorrecting capabilities and fixes the diffusional broadening existing in other commercial instruments.
3. 3D numerical simulations for single particle trajectories using stochastic diffusion in SIMION for the IDT are used to obtain resolutions of ions and separation ratios. Intermittent Push Flow resolutions acquired agree qualitatively with those predicted analytically. For Nearly-Stopping Potential Separation, the modeling of the instrument is shown to be able to separate particles of 55.89 and 55.93 nm with ease. This would require effective resolutions of several thousands.
4. A chromatography existing concept of resolving power is used to differentiate between peak resolution in the IDT and acceptable separation between similar mobility sizes (resolving power). It is shown that the IDT has a theoretically high resolving power for both intermittent push flow and nearly-stopping potential separation.
